# Rapid response and surveillance for high-risk contacts during May 2026 Andes hantavirus outbreak: a Northern Italy experience

**DOI:** 10.3389/fpubh.2026.1907655

**Published:** 2026-08-05

**Authors:** Sara Laura Ferrari, Stefano Odelli, Marta Colaneri, Alice Baratelli, Simone Di Stefano, Luca Meroni, Davide Mileto, Alessandro Mancon, Angela Ancona, Sabrina Senatore, Anna Carole D’Amelio, Marino Faccini, Luigi Vezzosi, Gabriele del Castillo, Antonio Piralla, Raffaele Bruno, Danilo Cereda, Fausto Baldanti, Andrea Gori, Collaborating Centre Study Group on Hantavirus

**Affiliations:** 1 Department of Infectious Diseases, Luigi Sacco University Hospital, ASST Fatebenefratelli-Sacco, Milan, Italy; 2 Department of Biomedical and Clinical Sciences, University of Milan, Milan, Italy; 3 Centre for Multidisciplinary Action for Global Health (MAGH), University of Milan, Milan, Italy; 4 Medical Direction Unit, Luigi Sacco University Hospital, ASST Fatebenefratelli-Sacco, Milan, Italy; 5 National PhD Programme in One Health Approaches to Infectious Diseases and Life Science Research, Department of Public Health, Experimental and Forensic Medicine, University of Pavia, Pavia, Italy; 6 Regional Center for Infectious Diseases (CeReMI), Milan, Italy; 7 Department of Food, Environmental and Nutritional Sciences (DeFENS), University of Milan, Milan, Italy; 8 Laboratory of Clinical Microbiology, Virology, and Bioemergencies, Luigi Sacco University Hospital, ASST Fatebenefratelli-Sacco, Milan, Italy; 9 Directorate General for Health of the Lombardy Region, Milan, Italy; 10 Agenzia di Tutela della Salute (ATS) Milano, Milan, Italy; 11 Department of Microbiology and Virology, Fondazione IRCCS Policlinico San Matteo, Pavia, Italy; 12 Department of Clinical-Surgical, Diagnostic and Pediatric Sciences, University of Pavia, Pavia, Italy; 13 Infectious Diseases Unit I, Fondazione IRCCS Policlinico San Matteo, Pavia, Italy

**Keywords:** hantavirus, high consequence infectious diseases, outbreak response, preparedness, public health

## Abstract

In May 2026, an outbreak of Andes hantavirus (ANDV) linked to the cruise ship MV Hondius prompted international contact tracing across multiple countries. Although population-level risk was low, the severity of hantavirus cardiopulmonary syndrome and limited evidence on person-to-person transmission supported precautionary management of selected contacts. Lombardy Region, Northern Italy, was notified of a contact-tracing chain involving four individuals: two direct flight-related contacts exposed to confirmed ANDV cases during international air travel and their two accompanying close contacts. A coordinated response was activated through regional, national, and international public health authorities. All four were referred to Luigi Sacco University Hospital and managed under precautionary measures proportionate to their exposure category. RT-PCR testing for ANDV was negative in all four. This experience illustrates how imported pathogens may generate complex regional demands even when population-level risk remains low, and underscores the value of predefined preparedness frameworks.

## Introduction

In May 2026, the World Health Organization (WHO) was notified of an outbreak of severe respiratory illness linked to the cruise ship MV Hondius. The vessel carried 147 passengers and crew members from 23 countries (1). As of 13 May 2026, 11 cases had been reported, including eight laboratory-confirmed Orthohantavirus andense (ANDV) infections, two probable cases, and one inconclusive result, with three deaths. The outbreak was characterized by severe clinical presentations, rapid deterioration in infected individuals, and the need for coordinated international public health action.

Between 12 and 18 May 2026, Lombardy Region was notified of a high-risk contact identified through the international investigations. The individual had been seated next to a passenger with laboratory-confirmed ANDV infection during an international flight. The index case had acquired the infection during the cruise ship outbreak and subsequently died. The initial notification concerned one direct flight-related contact. During the subsequent investigation, the regional response pathway was extended to three additional persons connected to the same contact-tracing chain: the accompanying close contact of the initially notified individual, a second direct flight-related contact exposed to the same confirmed case during a subsequent flight, and the accompanying close contact of the second direct contact. Following contact tracing, the two direct flight-related contacts and their accompanying close contacts were assessed through the regional pathway and referred to *Luigi Sacco* University Hospital, Milan, for clinical and public health management. Although international risk assessments considered the risk for the general population to be low, the severity of associated hantavirus cardiopulmonary syndrome (HCPS) and the limited evidence on ANDV human-to-human transmission supported a precautionary response (2, 3). Italian and regional health authorities therefore activated procedures for quarantine, active monitoring, rapid clinical assessment, and laboratory evaluation of individuals classified as high-risk contacts.

This article describes the rapid response model implemented in Lombardy Region for the assessment and management of high-risk contacts following exposure to a confirmed ANDV case. Although not fully generalizable, this experience may support preparedness planning for rare imported infections with potential person-to-person transmission.

## Governance and surveillance framework

The infectious disease surveillance and rapid response system in Lombardy is based on continuous coordination between regional, national, and international public health authorities. During health emergencies, this system operates through two complementary pathways.

The first is a top-down pathway. Lombardy Region maintains direct communication with the Italian Ministry of Health, which in turn receives updated information from the WHO and international alert and response systems regarding confirmed cases, outbreak evolution, and potential high-risk contacts entering the Italian territory.

The second is a bottom-up pathway. Territorial surveillance is coordinated through the eight Local Health Protection Agencies (Agenzie di Tutela della Salute, ATS), which oversee notifications of suspected infectious diseases reported by physicians across the region through the regional infectious diseases notification platform (SMI) (4, 5).

The regional governance framework is supported by the Regional Center for Infectious Diseases (Centro Regionale Malattie Infettive, CeReMI), established by Lombardy Region as a collaborative platform between ASST Fatebenefratelli-Sacco, Milan, and Fondazione IRCCS Policlinico San Matteo, Pavia (6). CeReMI integrates clinical, microbiological, epidemiological, and public health expertise. During the response phase, CeReMI provides technical and operational support to the Directorate General for Welfare of Lombardy Region, in close and continuous coordination with the regional Prevention Unit.

Luigi Sacco University Hospital (part of ASST Fatebenefratelli-Sacco) serves as a major regional referral center for infectious diseases and biological threats, and Fondazione IRCCS Policlinico San Matteo contributes to preparedness activities and specialized diagnostic capacity. Together, the two focal points of CeReMI perform analyses in parallel, ensuring operational redundancy and independent confirmation of results.

## Case notification and activation of the response

On 12 May 2026, an international alert concerning a British citizen in Italy, subsequently identified as the first of the two direct flight-related contacts managed in this response, was transmitted by United Kingdom authorities to the Italian Ministry of Health and the Ministry of the Interior. The notification activated the institutional coordination pathway involving the Prefecture, Lombardy Region, CeReMI, and ATS.

The initially notified individual had traveled, within the preceding 17 days, on the same international flight as two subsequently confirmed ANDV cases linked to the MV Hondius. Subsequent epidemiological investigations indicated that at least one of the two cases died shortly after the flight. The exposure occurred during a 4-h and 45-min flight from Saint Helena to Johannesburg. This close contact was seated one row from one case and three rows from the other. Given the flight duration, seating proximity, the severity of the cases, and the limited evidence on person-to-person transmission of Andes virus during air travel, the exposed passenger was managed conservatively and classified as a high-risk contact according to the precautionary principle. Regarding the second close contact, the exposure had occurred during a flight from Johannesburg to Amsterdam 24 days before notification. Although the UK authorities did not provide information on the clinical status of the Andes virus case or the seating arrangement, epidemiological reconstruction indicated that the case had attempted to board the Amsterdam flight before being denied boarding and subsequently died shortly afterwards. Given the limited information available and the uncertainty surrounding person-to-person transmission of Andes virus during air travel, the exposed passenger was managed conservatively and classified as a high-risk contact according to the precautionary principle.

Following activation by Lombardy Region, the hospital health management and medical direction of ASST Fatebenefratelli Sacco coordinated the operational response. The bioemergency response team was mobilized, and ATS was involved to support territorial public health surveillance and the management of any potential community implications.

At the time of notification, the initially identified direct contact had not yet been informed of his epidemiological status and was freely circulating in the community. Once identified, the direct flight-related contacts and their accompanying close contacts were referred to the Infectious Diseases Unit of Luigi Sacco University Hospital and managed under precautionary measures proportionate to their exposure category.

Regarding clinical symptoms, based on the epidemiological investigations and clinical assessments, only the first identified close contact reported symptoms. Symptom onset occurred 10 days after the potential exposure during the flight and 7 days before the contact was identified. The reported symptoms included gastrointestinal manifestations, cough, and rhinorrhea.

The contact was tested on the same day that the UK authorities notified the Italian authorities of their identification as a close contact. Testing for Andes virus was performed immediately and yielded a negative result; however, respiratory testing was positive for rhinovirus and detected a low bacterial load of *Streptococcus pneumoniae*. A second test was performed 72 h after the contact had been identified as a close contact following a single episode of fever, and the result was also negative for Andes virus.

One traveling companion (a secondary contact) remained asymptomatic but was also tested for Andes virus, with a negative result, and was found to be positive for a low bacterial load of *Streptococcus pneumoniae*: quarantine was not implemented for this secondary contact.

The second close contact, notified to the Italian authorities by the UK authorities 24 days after the last potential exposure, remained asymptomatic throughout the follow-up period and was tested both at the beginning and at the end of the quarantine period. Both tests were negative for Andes virus.

In this case, an asymptomatic travel companion (secondary contact) was also identified. The individual tested negative for Andes virus, and quarantine was not implemented.

To minimize the potential risk of transmission during hospital assessment and management, infection prevention and control (IPC) measures were strictly implemented, including the systematic use of personal protective equipment (PPE) and strict hand hygiene. Recommendations for healthcare workers included the systematic application of standard precautions combined with droplet precautions during routine patient care and close contact activities. Environmental hygiene and sanitation measures were reinforced according to institutional protocols, with procedures designed to minimize the risk of aerosolization of contaminated particles.

Virological assessment was performed according to exposure category and clinical indication using nasopharyngeal swab, blood, and urine specimens. Based on a biological risk assessment conducted for laboratory personnel and considering that orthohantaviruses are classified as Risk Group 3 agents, all primary specimens were initially handled in a biosafety level 3 (BSL-3) laboratory within a Class II biological safety cabinet (BSCII) by staff wearing dedicated personal protective equipment. Nucleic acid extraction was performed entirely under BSL-3 conditions. Subsequent molecular analyses were carried out in a biosafety level 2 (BSL-2) laboratory using extracted nucleic acids. RealTime-PCR assays performed at Luigi Sacco University Hospital were negative for ANDV in all four individuals, with rhinovirus detected in the first evaluated contact. Metagenomic investigations performed at Fondazione IRCCS Policlinico San Matteo confirmed the absence of ANDV in all samples and the presence of rhinovirus in the first evaluated contact.

Public communication was managed as part of the overall response strategy. Communication activities were coordinated between the hospital communication office, Lombardy Region, and the Italian Ministry of Health. To ensure consistency and avoid conflicting public messages, the first official communication to the media was centrally released by the Ministry of Health (7, 8) followed by regional institutional communication and subsequent technical clarifications provided by the hospital communication office (9).

**Figure 1:**
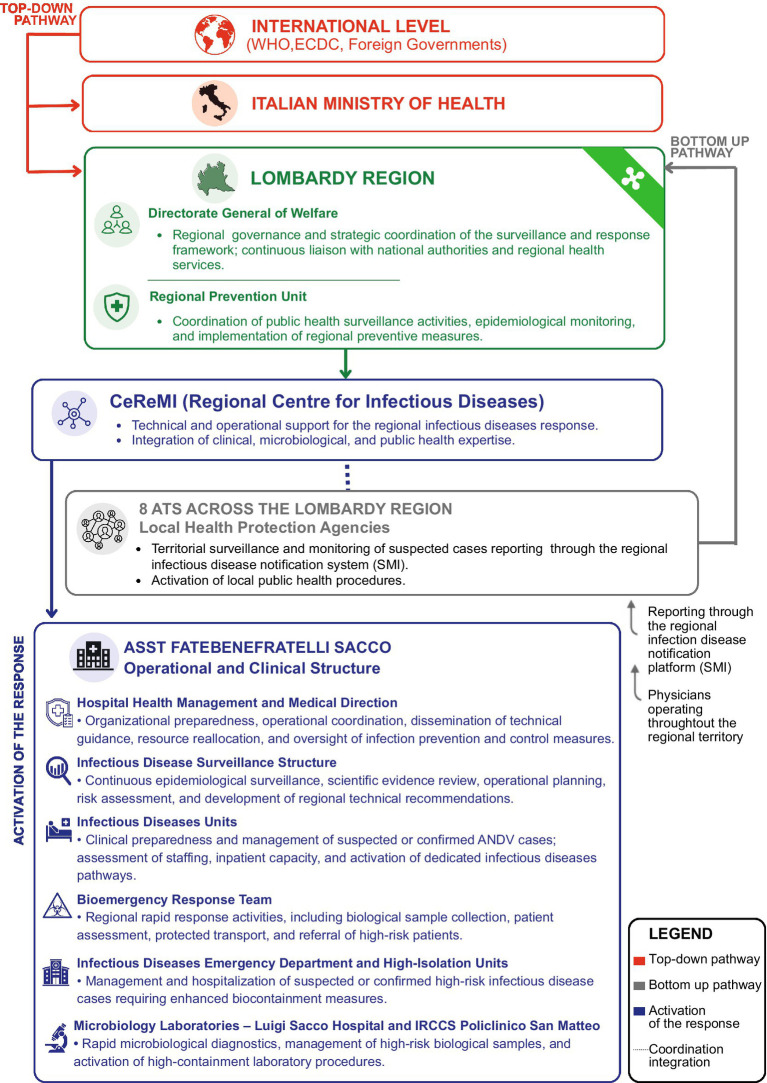
Governance and rapid response framework for the management of high-risk contacts during the ANDV outbreak, Lombardy Region, Italy, May 2026.

The response relied on the coordinated action of multiple institutions and operational structures (Figure 1).

## Discussion

The notification of a small contact-tracing chain in Lombardy illustrates how an outbreak occurring outside Europe and assessed as posing a very low risk to the general EU/EEA population, may still require a rapid and coordinated local public health response. In highly connected travel settings, individuals with relevant exposure histories may enter the community before local authorities have received sufficient information to assess risk and define appropriate measures. The main operational challenge is therefore to act while information is still evolving, while ensuring the response remains proportionate to the actual epidemiological risk and exposure category. Human-to-human transmission is rare (10), and has mainly been described after close and prolonged exposure (11), while HCPS may have a severe and rapidly progressive clinical course (12). This combination supports a precautionary approach for individuals classified as high-risk contacts, particularly during the incubation period. In addition, the incubation period may extend up to 6–8 weeks, complicating decisions regarding the duration and proportionality of quarantine and active monitoring (13). In this event, the first two identified contacts had traveled extensively within Italy for approximately 2 weeks before being identified following an international public health notification transmitted through the International Health Regulations (IHR) communication mechanism. The notification was issued by the United Kingdom National IHR Focal Point and received by its Italian counterpart, informing the national authorities of the presence of potentially exposed contacts on Italian territory. The Italian Ministry of Health subsequently alerted the competent Regional Health Authorities, activating the regional surveillance and response system. Within this scheme, operational coordination and contact-related information exchange were managed bilaterally between the Italian and United Kingdom IHR Focal Points, while ECDC and WHO acted in a supporting and advisory capacity rather than as coordinating bodies. The event was additionally shared with EU/EEA Member States through the Early Warning and Response System (EWRS) operated by ECDC. This configuration illustrates how delays in international notification and contact tracing may allow potentially exposed individuals to circulate widely before local containment measures can be implemented.

At the same time, the rarity of transmission requires careful targeted rather than broad public health measures. Containment actions should be guided by necessity and proportionality, with their intensity and duration linked to exposure category, incubation period, clinical risk, and evolving evidence on transmissibility (14, 15). No retrospective community contact tracing covering the individuals’ travel period, nor public health alerts to healthcare facilities along their itinerary, were undertaken. This decision was consistent with the official note issued by the Ministry of Health: although the two individuals were classified as contacts of a confirmed case, documented person-to-person transmission of Andes virus is associated with close contact with symptomatic cases, particularly during the cardiopulmonary phase. Both individuals did not meet the case definition for confirmed ANDV infection, and subsequent laboratory testing returned negative results; the risk of onward community transmission was therefore considered negligible. Rather than issuing broad facility-level notifications, the two contacts were placed under active surveillance for the full duration of the incubation period, and their travel itinerary within Italy was reconstructed to enable the rapid, targeted implementation of additional measures in the event of laboratory confirmation or compatible clinical evolution. Accordingly, direct flight-related contacts, in accordance with national-level public health guidance based on WHO and ECDC documents (16, 17), were quarantined for 42 days and actively monitored throughout the incubation period, while accompanying close contacts without direct exposure to the confirmed case did not meet the risk criteria for ANDV exposure, given that documented person-to-person transmission requires close contact with a symptomatic case; they were therefore not classified as at-risk contacts and were not subject to quarantine or active public health follow-up. Predefined preparedness pathways are essential to translate precaution into timely but proportionate action (18). In this event, the availability of defined institutional responsibilities, referral routes, transport procedures, laboratory capacities, and communication channels reduced the time between notification and operational response. For rare, imported infections with potentially severe outcomes, preparedness is most valuable when it enables health authorities to act before uncertainty becomes urgency. The event also highlighted the difficulty of maintaining readiness between alerts: preparedness should be conceived as a scalable system, anchored in routine surveillance and clinical activity but capable of rapid expansion, to prevent the erosion of capabilities that are rarely used but essential during biological emergencies.

Importantly, the value of such pathways should not be limited to highly specialized referral facilities. Establishing rapid response mechanisms in a broader range of clinical settings can strengthen the overall system’s capacity to absorb the initial response phase. Risk communication should be an integral component of preparedness. Conflicting or premature messages from different institutions may amplify uncertainty (19). In this event, communication was coordinated between Luigi Sacco Hospital, Lombardy Region, and the Ministry of Health, and public information was released only after the individuals under assessment had been identified and appropriate precautionary measures had been activated. This approach helped clarify that monitoring and isolation were targeted risk-management measures, rather than evidence of wider community transmission (20).

Regional surveillance systems can help sustain preparedness over time. Positioned between territorial services and central authorities, they can recognize signals arising locally and frame them within national and international alert structures, a role particularly relevant for imported infections, where the challenge is often not widespread transmission but the rapid assessment of individual exposures and coordination of an appropriate response across the health system.

## Lessons learned

This experience highlights several operational lessons. First, international information sharing must be rapid enough to allow local health authorities to identify exposed individuals before prolonged community circulation occurs; delays can complicate management even when the overall risk to the general population is low. Second, rare imported infections require predefined response pathways. These pathways should define governance, referral routes, laboratory responsibilities, and communication chains before an event occurs. Third, proportionality is essential: precautionary measures for selected individuals should remain targeted and linked to exposure category, clinical risk, and the evolving evidence base. Fourth, preparedness cannot rely only on highly specialized centers. Referral centers remain essential, but rapid response mechanisms should also be understandable and partially implementable in broader clinical settings, which may be the first point of contact for exposed or symptomatic individuals.

Retrospectively, some aspects of the response highlight room for improvement. The interval between the exposure event and its notification meant that both contacts had already traveled extensively within Italy before being identified; this reflects the time required for international detection and reporting chains rather than a delay in the regional response itself, but it underscores the value of faster data exchange between IHR National Focal Points for travel-related exposures identified after the event has occurred. Similarly, the absence of a standardized minimum dataset for cross-border contact notifications, evident here in the incomplete information available on the clinical status of the source case and on seating arrangements for one contact, limited the precision of risk stratification and likely contributed to a more conservative classification than might otherwise have been warranted. Finally, coordination between the two countries relied on *ad hoc* bilateral exchange rather than a pre-agreed rapid-response protocol; embedding such a protocol within existing IHR and EWRS channels could shorten response times in future events without reducing the flexibility needed to manage atypical presentations.

Finally, communication should be planned as carefully as clinical and laboratory procedures. Coordinated institutional messaging can maintain public trust, reduce unnecessary alarm, and explain why restrictive measures may be appropriate for selected contacts despite a low risk for the wider population.

## Conclusion

The management of high-risk contacts during the ANDV outbreak demonstrates that imported pathogens can generate complex public health demands at the regional level, even when the risk for the general population remains low. In this setting, a coordinated and predefined preparedness framework enabled a rapid, targeted, and proportionate response. The central challenge for regional health systems is not the capacity to respond when an event is already unfolding, but the ability to maintain that capacity during the intervals between alerts, when the pressure is absent but the need for readiness remains. Investing in preparedness as a continuous function, rather than an emergency reaction, is what ultimately determines the quality of the response when it is needed.

## Data Availability

The datasets presented in this article are not readily available because the original contributions presented in the article are included in the article. Additional individual-level data are not publicly available due to privacy and public health confidentiality requirements. Further inquiries can be directed to the corresponding author, where appropriate and in accordance with applicable regulations. Requests to access the datasets should be directed to Stefano Odelli, stefano.odelli01@universitadipavia.it.

## Members of the collaborating centre study group on hantavirus

Davide Bernasconi, Monica Schiavini, Ivano Faggion, Marco Schiuma, Juliana Viazzo, Alberto Dolci, Alberto Rizzo, Simone Villa, Manuel Maffeo, Emanuele De Ponti, Federica Attanasi, Maria Giulia Abbruzzese, Ilaria Pucci, Francesca Longhi, Danila Caso, Benedetta Andreoni, Luana Giannino, Elisa Buttinelli, Francesca Rovida.
